# Dr. Sherbino’s Clinical Cases in August Number

**Published:** 1887-10

**Authors:** 


### DR. SHERBINO'S CLINICAL CASES IN THE AUGUST NUMBER.

By an astonishing oversight upon the part of the editor in reading proof, the remedies for Cases II and III were omitted. (Page 302.)

In Case II the remedy was Lilium tig.$^{40m}$. In Case III the remedy was Bell.$^{cm}$.

The absence of this important information makes nonsense of these instructive cases. W. M. J.

